# Clinical outcomes and prognostic factors after first- and second-line immunotherapy in a cohort of 57 patients with refractory autoimmune encephalitis

**DOI:** 10.3389/fimmu.2026.1907553

**Published:** 2026-09-30

**Authors:** Jiao He, Haozhe Lu

**Affiliations:** 1 Department of Neurology, The First Affiliated Hospital of Zhengzhou University, Zhengzhou, Henan, China; 2 Healthcare Security Administration Office, The First Affiliated Hospital of Zhengzhou University, Zhengzhou, Henan, China

**Keywords:** autoimmune encephalitis, plasma cell-depleting therapy, real-world study, refractory autoimmune encephalitis, timing of immunotherapy

## Abstract

**Background:**

Refractory autoimmune encephalitis (AE) is a severe condition with limited real-world evidence on treatment outcomes and prognostic factors. This study evaluated clinical outcomes and factors associated with 6-month functional recovery in a strictly defined refractory AE cohort.

**Methods:**

This single-center retrospective cohort study screened 246 potentially eligible AE records from 2018 to 2025 and included 57 patients who fulfilled the prespecified refractory AE criteria and had ascertainable 6-month functional outcomes. Second-line immunotherapy was subsequently administered to a subset of patients. The primary outcome was 6-month functional status assessed by the modified Rankin Scale (mRS) after refractory-state adjudication, with favorable outcome defined as mRS 0–2. Multivariable logistic regression, adjusted for baseline severity and constrained by the limited number of unfavorable outcomes, examined factors associated with 6-month functional outcome.

**Results:**

All 57 patients fulfilled the harmonized refractory criterion after inadequate response to first-line immunotherapy. Second-line escalation was subsequently administered to 14 patients (24.6%), whereas 43 (75.4%) did not receive second-line therapy. Favorable 6-month outcomes occurred in 9/14 (64.3%) and 28/43 (65.1%), respectively.

**Conclusions:**

In the parsimonious multivariable analysis, higher mRS at refractory-state adjudication and ICU admission were associated with unfavorable 6-month functional outcome. The association with ICU admission persisted after adjustment for mRS, but its magnitude was imprecisely estimated and likely overlaps with the underlying construct of disease severity. These findings should therefore be considered exploratory prognostic associations rather than definitive independent predictors.

## 1 Introduction

Autoimmune encephalitis (AE) is an immune-mediated disorder of the central nervous system whose recognition has grown substantially. It typically presents with acute/subacute seizures, psychiatric symptoms, cognitive impairment, and reduced consciousness, and may rapidly lead to severe disability. Nationwide data show that the incidence of AE and related paraneoplastic syndromes rose from 4.70 to 5.76 per million person-years between 2016 and 2021, with anti-NMDAR, anti-LGI1, anti-Hu, and anti-GAD65 accounting for nearly two-thirds of cases (1). A population-based study reported a prevalence of 13.7 per 100,000, approaching that of infectious encephalitis, indicating AE is an increasingly important public health problem rather than a rare diagnosis (2). In children the burden is likely underestimated (3).

AE is clinically important not only because it can cause seizures, psychiatric manifestations, impaired consciousness, autonomic dysfunction, and respiratory failure (4), but also because timely immunotherapy greatly improves outcomes. Current treatment follows a stepwise approach: first-line therapy includes high-dose corticosteroids, intravenous immunoglobulin, and plasma exchange; second-line therapy mainly comprises rituximab or cyclophosphamide (5). Nevertheless, many patients have persistent seizures, cognitive deficits, fatigue, sleep disturbance, and mood symptoms after the acute phase, and long-term follow-up of anti-NMDAR encephalitis shows memory, language, and social deficits may last for years (6). Delayed immunotherapy is associated with poor long-term cognition (7), and diagnostic uncertainty, especially in children, complicates treatment escalation (3).

AE is driven by humoral and cellular immunity as well as cerebral microenvironment abnormalities (8, 9). Antibodies against neuronal surface antigens cause receptor internalization, synaptic disruption, and network imbalance, while B cells, T cells, and cytokine networks contribute to disease persistence and treatment resistance (8, 9). Some patients fail to achieve meaningful remission after initial immunotherapy and develop refractory disease, defined by prolonged neuropsychiatric impairment, recurrent status epilepticus, or continued dependence on neurocritical care (10, 11). Current guidelines recommend timely escalation to second-line therapy when first-line response is inadequate, and maintenance immunotherapy based on subtype, relapse risk, and clinical course (4, 5). However, evidence for refractory disease is limited, so treatment selection remains variable and must balance efficacy, safety, and accessibility (10, 12).

The literature has two critical gaps. First, real-world data on cumulative outcomes of heterogeneous treatment pathways (first-line, second-line, and maintenance) in strictly defined refractory cohorts are scarce (10, 12). Second, short-term functional recovery in severe refractory AE likely reflects a complex interaction between baseline neurological severity and secondary complications from intensive care (11, 13). ICU admission and mechanical ventilation are often life-saving, but prolonged neurocritical care may bring pulmonary infection, immobility, and other systemic complications that adversely affect recovery (11, 13). Thus, determining the independent prognostic value of baseline characteristics, antibody subtype, CSF/blood–brain barrier biomarkers, and critical care needs remains a major challenge (14–16).

In this single-center retrospective real-world cohort of 57 patients with refractory AE, we systematically characterized clinical profiles and chronological immunotherapy pathways. Furthermore, by controlling for baseline clinical heterogeneity, we evaluated the independent relationships between critical care requirements, antibody status, CSF findings, blood–brain barrier dysfunction, and 6-month functional outcomes. This study aims to provide methodologically adjusted clinical evidence to optimize stepwise immunotherapy selection and critical care management in refractory AE.

## 2 Materials and methods

### 2.1 Study population

#### 2.1.1 Study design

This single-center retrospective cohort study used routinely collected real-world clinical data. It aimed to characterize clinical features, immunotherapy pathways, and short-term outcomes in refractory AE and to identify prognostic factors. The primary focus was the relationship between demographic characteristics, disease severity, immunotherapy strategies, critical care requirements, antibody status, cerebrospinal fluid (CSF) findings, blood–brain barrier (BBB) dysfunction, and short-term functional outcome.

Eligible patients fulfilled the diagnostic criteria for AE and met a prespecified definition of refractory disease after an inadequate clinical response to adequate first-line immunotherapy. Second-line immunotherapy was considered a subsequent escalation strategy after refractory status had been established and was not required for cohort eligibility. Maintenance immunosuppression was recorded separately as a relapse-prevention or longer-term disease-control strategy and was not used to define treatment refractoriness. Because no universally accepted definition exists, refractory disease was defined according to an institutional protocol based on published literature, clinical consensus, and local neuroimmunology practice. The analytic population was restricted to patients with sufficient core clinical, treatment-response, refractory-state adjudication, and 6-month outcome data; complete information on all exploratory laboratory, imaging, or EEG variables was not required.

#### 2.1.2 Study period and source population

Patients admitted to our hospital between January 1, 2018, and December 31, 2025, were included. The center provides specialized neuroimmunology and neurocritical care services and maintains structured electronic records for AE patients.

Potentially eligible patients were identified from inpatient electronic medical records, discharge summaries, neurocritical-care records, and follow-up databases. During the study period, 246 records with a discharge diagnosis or clinically suspected diagnosis of autoimmune encephalitis were screened. Diagnostic eligibility was reassessed by investigator review. Forty-eight records were excluded at this stage, including 31 in which the final diagnosis was revised to a non-autoimmune neurological or systemic disorder and 17 with insufficient evidence to satisfy definite or probable AE criteria. The remaining 198 patients constituted the diagnostically eligible AE population.

Among these patients, 102 showed an adequate response to first-line immunotherapy or otherwise did not fulfill the prespecified refractory AE definition and were excluded. Ninety-six patients therefore underwent detailed refractory-cohort eligibility review. A further 39 were excluded because of a major competing systemic condition affecting outcome interpretation (n = 12), insufficient key clinical or treatment data (n = 14), unavailable 6-month functional outcome despite follow-up tracing (n = 8), or duplicate/recurrent episodes for which another hospitalization was selected as the predefined index episode (n = 5). The final analytic cohort therefore comprised 57 patients with refractory AE.

### 2.2 Cohort eligibility and index-episode selection

#### 2.2.1 Diagnostic eligibility

Patients were diagnostically eligible if they were aged ≥14 years and fulfilled investigator-confirmed definite or probable AE criteria based on the overall clinical syndrome together with available CSF, EEG, brain MRI, neuronal-antibody, and other etiologic investigations. Alternative infectious, metabolic, toxic, primary psychiatric, neurodegenerative, or other non-autoimmune causes were excluded through clinical review.

Tumor screening was incorporated into the etiologic assessment when clinically indicated, particularly when the clinical phenotype or antibody profile raised concern for paraneoplastic AE. A positive tumor finding was not required for inclusion, and a negative tumor screen was not itself an eligibility criterion. Rather, tumor-related information was used to support etiologic classification and to distinguish autoimmune/paraneoplastic encephalitis from direct neoplastic involvement of the central nervous system.

#### 2.2.2 Refractory AE definition and index-episode selection

Eligible patients were required to have received adequate first-line immunotherapy during the active AE episode and to subsequently fulfill the prespecified refractory AE definition, namely persistent or progressive neurological dysfunction despite adequate first-line treatment. Receipt of second-line or maintenance immunotherapy was not required for eligibility.

For patients with multiple hospitalizations during the same disease course or more than one episode potentially satisfying the refractory criteria, only one index episode was included. The index episode was defined as the earliest hospitalization or active disease episode during which the patient first fulfilled the prespecified refractory AE criteria after inadequate response to first-line immunotherapy. Subsequent admissions, relapses, and repeated records from the same patient were not treated as independent observations.

#### 2.2.3 Minimum follow-up and data requirements

Patients were required to have sufficient documentation to establish the timing of first-line treatment, refractory-state adjudication, baseline mRS at refractory-state adjudication, and the primary 6-month functional outcome. A minimum of 6 months of outcome ascertainment was required through outpatient records, rehospitalization records, rehabilitation documentation, or structured telephone follow-up. Complete information on every exploratory biomarker or secondary variable was not required; however, cases were excluded when missing information prevented reliable confirmation of AE diagnosis, refractory status, index-episode selection, or the primary 6-month mRS outcome.

#### 2.2.4 Predefined exclusions

Patients were excluded for any of the following predefined reasons:

1. Diagnostic ineligibility: the final diagnosis was revised to a non-autoimmune neurological disorder or available evidence was insufficient to establish definite/probable AE.
2. Failure to meet refractory AE criteria: adequate clinical improvement after first-line immunotherapy or absence of documented persistent/progressive neurological dysfunction required for refractory-state adjudication.
3. Major competing systemic condition: a concurrent systemic illness was considered competing when it independently required intensive treatment and was expected to dominate short-term survival or functional outcome irrespective of AE. This included severe active infection with sepsis, septic shock, or infection requiring ICU-level organ support; severe hepatic failure with clinically evident hepatic decompensation or marked impairment of synthetic function; severe renal failure requiring renal-replacement therapy or equivalent advanced organ support; progressive malignancy with major short-term prognostic impact; or decompensated cardiopulmonary disease requiring advanced organ support.
  Severe active infection was defined as microbiologically or clinically established infection accompanied by sepsis/septic shock or infection-related organ dysfunction requiring ICU-level organ support before or independently of the refractory AE course. Nosocomial pulmonary infection occurring as a complication of AE-related intensive care was not an exclusion criterion.
  Severe hepatic failure was defined as clinically decompensated hepatic dysfunction with impaired synthetic function and/or hepatic encephalopathy considered sufficient to independently influence short-term prognosis. Isolated transaminase elevation without hepatic decompensation was not an exclusion criterion.
  Severe renal failure was defined as renal dysfunction requiring renal-replacement therapy or advanced organ support and judged to independently dominate short-term prognosis. Mild or transient acute kidney injury not requiring renal-replacement therapy was not an exclusion criterion.
4. Inadequate core data or follow-up: inability to reliably determine first-line treatment exposure, refractory-state adjudication, baseline mRS, index episode, or 6-month functional outcome despite review of available records and follow-up tracing. Duplicate admissions or recurrent episodes were excluded when they represented the same patient after selection of the predefined index episode.

### 2.3 Definition of refractory disease, outcome grouping, and treatment pathways

#### 2.3.1 Definition and adjudication of refractory autoimmune encephalitis

All potentially eligible cases underwent systematic medical-record review before inclusion. After confirmation of the AE diagnosis according to the predefined diagnostic framework, investigators reviewed the clinical course and response to first-line immunotherapy to determine whether the prespecified refractory criteria were fulfilled.

Refractory autoimmune encephalitis was operationally defined as persistent or progressive neurological dysfunction despite adequate first-line immunotherapy during the active AE episode. Adequate first-line therapy comprised high-dose corticosteroids, intravenous immunoglobulin, plasma exchange, or an appropriate combination of these modalities, as clinically indicated. Clinical response was assessed after completion or adequate delivery of the intended first-line regimen. Persistent or progressive neurological dysfunction was defined by one or more of the following: absence of meaningful functional improvement compared with the pre-treatment or post-first-line status; persistent impairment of consciousness; marked psychiatric or behavioral disturbance; ongoing seizures or status epilepticus; continued requirement for intensive care or mechanical ventilation because of active neurological disease; or persistent inflammatory abnormalities accompanied by clinical non-improvement or deterioration.

The date on which these criteria were first fulfilled after adequate first-line therapy was recorded as the refractory-state adjudication date and served as the common time zero for all patients. Second-line immunotherapy was considered a subsequent escalation strategy after refractory-state adjudication and was not required for cohort eligibility. Accordingly, patients who subsequently received second-line therapy and those who did not entered follow-up from the same clinically defined disease stage.

All 57 included cases were re-reviewed using this harmonized definition. All 57 patients (100%) fulfilled the refractory AE criteria after inadequate response to first-line immunotherapy. Subsequently, 14/57 (24.6%) received second-line immunotherapy and 43/57 (75.4%) did not undergo second-line escalation. Maintenance immunosuppression was administered to 10/57 patients (17.5%) as a longer-term disease-control or relapse-prevention strategy and was not used to define refractoriness.

#### 2.3.2 Definition of functional outcome groups

Patients were classified into favorable- and unfavorable-outcome groups according to their 6-month modified Rankin Scale scores. A favorable functional outcome was defined as an mRS score of 0–2, whereas an unfavorable functional outcome was defined as an mRS score of 3–6. Six months after refractory-state adjudication was selected as the primary outcome-assessment time point. Refractory-state adjudication, defined by inadequate response to first-line immunotherapy, served as the common time zero for all patients irrespective of whether second-line therapy was subsequently administered. Accordingly, the 6-month outcome was assessed at a comparable disease stage in the second-line and non-second-line groups rather than from the date of second-line treatment initiation.

Six-month mRS scores were determined from outpatient records, rehospitalization records, rehabilitation assessments, or structured telephone follow-up. When information differed across sources, the assessment closest to the prespecified 6-month time point and supported by the most complete clinical documentation was used.

Treatment pathways were classified only after refractory-state adjudication. Second-line immunotherapy represented escalation for persistent active disease after first-line treatment, whereas maintenance immunosuppression represented longer-term disease control or relapse-prevention management. Maintenance therapy therefore did not contribute to the operational definition of refractory AE.

No additional qualitative categories such as “good,” “fair,” or “poor” were used for the primary or secondary functional outcome classification; functional outcome was reported consistently according to the prespecified mRS-based definition.

#### 2.3.3 Reconstruction and classification of immunotherapy pathways

To characterize treatment trajectories, each patient’s clinical course was reconstructed chronologically and classified into three immunotherapy categories: (1) first-line immunotherapy; (2) second-line immunotherapy; and (3) maintenance immunosuppression. Supportive neurological and critical-care treatments were recorded separately and were not classified as immunotherapy.

First-line immunotherapy included high-dose corticosteroids, intravenous immunoglobulin, plasma exchange, or combinations thereof. Second-line immunotherapy included rituximab, cyclophosphamide, or ofatumumab. Maintenance immunosuppression included mycophenolate mofetil or azathioprine and was considered a longer-term disease-control or relapse-prevention strategy rather than evidence of treatment failure. For cohort inclusion, the minimum required treatment information comprised the first-line regimen, treatment initiation date, sufficient documentation of clinical response, and the refractory-state adjudication date. Additional information on dose, treatment duration, number of cycles, repeated administration, and subsequent immunotherapy was extracted when documented but was not required for eligibility.

Repeated corticosteroid pulses, intravenous immunoglobulin, or plasma exchange were recorded as additional acute-phase immunotherapy. Antiseizure medication, mechanical ventilation, hemodynamic support, and other neurocritical-care interventions were classified separately as supportive treatment and were not considered components of the immunotherapy pathway.

#### 2.3.4 Variable collection and operational definitions

Data were extracted using a standardized case report form developed collaboratively by the study team and implemented uniformly. Extraction was performed by trained investigators in accordance with a predefined variable dictionary.

Collected variables included demographic characteristics, disease-specific features, markers of disease severity, and treatment-pathway variables. Demographic variables included age, sex, history of autoimmune disease, and tumor history. Disease-characteristic variables included antibody status and subtype, presenting symptoms, psychiatric or behavioral abnormalities, seizures, impaired consciousness, autonomic dysfunction, cerebrospinal fluid cell count and protein concentration, oligoclonal bands, intrathecal immunoglobulin synthesis, blood–brain barrier dysfunction, EEG abnormalities, and brain MRI abnormalities. Baseline neurological severity was anchored to the refractory-state adjudication date. Baseline mRS was defined as the mRS score documented at, or closest to and before, the date on which refractory AE was first adjudicated after inadequate response to first-line immunotherapy. This definition was applied uniformly irrespective of subsequent second-line treatment. Other clinical severity and critical-care variables included impaired consciousness, status epilepticus, autonomic dysfunction, ICU admission, mechanical ventilation, and hemodynamic support. Treatment-pathway variables included first-line treatment regimen, second-line treatment regimen, time from symptom onset to treatment initiation, and use of maintenance immunotherapy such as mycophenolate mofetil or azathioprine.

All time-related variables were calculated in calendar days. Symptom onset was defined as the date of the first neuropsychiatric symptoms attributable to the current AE episode. When the exact day was unavailable but the month of onset was documented, the 15th day of that month was used as a prespecified substitute date. This substitution was required in 8 of 57 patients (14.0%). Time-dependent variables derived from symptom onset in these patients were therefore considered approximate. All key variables underwent secondary review after data entry, and uncertain cases were rechecked against original records and clarified with the treating team when necessary.

#### 2.3.5 Follow-up procedures

All 57 patients entered follow-up from the refractory-state adjudication date, which was defined uniformly as the date on which inadequate response to adequate first-line immunotherapy was first documented. This common time zero was applied irrespective of subsequent second-line or maintenance treatment, thereby avoiding treatment-dependent differences in the starting point of outcome follow-up. Follow-up data were obtained from outpatient records, rehospitalization records, and structured telephone interviews. Investigators first reviewed follow-up visits within our center and recorded subsequent clinical assessments and functional changes. For patients who did not return for scheduled follow-up, missing information was supplemented by telephone follow-up.

Telephone interviews were conducted by uniformly trained investigators using a standardized protocol when an in-person 6-month assessment was unavailable. Functional status was reconstructed from structured questions addressing mobility, activities of daily living, dependence, and major neurological disability and was then mapped to the corresponding mRS category. When both clinical records and telephone information were available, the assessment closest to the prespecified 6-month time point and supported by the most complete documentation was used. Follow-up focused on neurological recovery, recurrence of neurological symptoms, and receipt of additional immunotherapy. All follow-up information was entered into a unified database and integrated with hospitalization records. If information obtained by telephone was clearly inconsistent with available medical records, further verification was sought through additional documentation review or family contact.

### 2.4 Outcomes

#### 2.4.1 Primary outcome

The primary outcome was functional status at 6 months after refractory-state adjudication, assessed using the modified Rankin Scale (mRS). The refractory-state adjudication date served as the uniform time zero for all patients and was determined after inadequate response to first-line immunotherapy, before classification according to subsequent second-line treatment exposure. Patients with an mRS score of 0–2 were classified as having a favorable outcome, indicating functional independence, while scores of 3–6 were considered unfavorable. The mRS ranges from 0 (no symptoms) to 6 (death) and is widely used in neurological disease studies. mRS scores were adjudicated by trained investigators using outpatient or rehabilitation documentation whenever available. When no contemporaneous in-person assessment was available, a structured telephone interview with the patient or an informed family member was used to reconstruct functional status. Because telephone-based mRS assessment may be less precise than direct clinical examination, this mode of ascertainment was considered a potential source of measurement variability.

#### 2.4.2 Secondary outcomes

Secondary outcomes included neurological improvement, ICU admission, duration of mechanical ventilation, length of hospital stay, relapse, mortality, and residual neurological or psychiatric deficits.

First clinical remission was defined as the interval from refractory-state adjudication to the first sustained clinical improvement, including recovery of consciousness, cessation or substantial reduction of seizures, stabilization of psychiatric or behavioral symptoms, and no further requirement for intensified immunotherapy. Relapse was defined as the reappearance or worsening of neuropsychiatric symptoms after initial remission, necessitating renewed immunotherapy and not better explained by infection, metabolic disturbance, or medication-related causes.

#### 2.4.3 Safety outcomes

Safety outcomes encompassed serious infection, hematologic toxicity, infusion- or allergy-related reactions, peripheral neuropathy, hepatic or renal injury, and treatment interruption due to adverse events. Adverse events were retrospectively graded according to the National Cancer Institute Common Terminology Criteria for Adverse Events (CTCAE), version 5.0 (17). Clinically significant infectious and hematologic adverse events were defined as CTCAE grade ≥3 whenever sufficient source information was available for retrospective grading. Hematologic events included grade ≥3 leukopenia, neutropenia, anemia, or thrombocytopenia. Infectious events included grade ≥3 infections requiring systemic antimicrobial treatment, hospitalization or prolongation of hospitalization, or other intensive medical management according to the applicable CTCAE category. Infusion- or allergy-related reactions included fever, chills, hypotension, dyspnea, rash, or similar events occurring during or shortly after drug administration that required medical intervention or treatment modification. Peripheral neuropathy was defined as clinically significant sensory or motor symptoms affecting daily activities or requiring specific therapy. Hepatic injury was defined as transaminase or bilirubin elevation exceeding three times the upper limit of normal, and renal injury as a substantial increase in serum creatinine or a marked decline in estimated glomerular filtration rate. Treatment interruption was defined as premature discontinuation or modification of first-line, second-line, or maintenance immunotherapy due to treatment-related adverse events.

This safety definition was distinct from the predefined exclusion criterion for severe active infection. The latter referred to infection already present before or independently of the refractory AE course and sufficiently severe to dominate short-term prognosis, whereas safety analyses captured infections occurring during or after treatment of the index refractory AE episode.

### 2.5 Statistical analysis

All statistical analyses were performed using Python 3.11.2 (pandas 2.2.3, SciPy 1.14.1, and statsmodels 0.14.3). Continuous variables are presented as mean ± SD for normally distributed data or median (IQR) for non-normally distributed data, and categorical variables as n (%). All tests were two-sided, with P < 0.05 considered statistically significant. Effect estimates are reported with 95% confidence intervals (CIs). Between-group comparisons used the Student’s t test or Mann–Whitney U test for continuous variables and the chi-square test or Fisher’s exact test for categorical variables.

Candidate factors were interpreted within a prespecified temporal and conceptual framework and grouped into four clinically defined domains: (1) demographic factors, including age and sex; (2) baseline disease characteristics and neurological severity at refractory-state adjudication, including mRS, antibody status/subtype, impaired consciousness, seizures, autonomic dysfunction, MRI/EEG findings, and CSF/BBB markers; (3) downstream critical-care markers, including ICU admission and mechanical ventilation; and (4) post-adjudication treatment-pathway variables, including second-line and maintenance immunotherapy. Treatment-related variables were considered exploratory because treatment allocation could be influenced by both disease severity and response to preceding therapy and were therefore not entered into the primary prognostic model.

Univariable logistic regression was used as an exploratory screening step to characterize the direction and magnitude of associations with unfavorable 6-month outcome rather than as an automatic stepwise variable-selection procedure. Variables with P < 0.10 were considered potentially informative, but multivariable inclusion additionally required clinical relevance, temporal appropriateness, acceptable completeness, and limited overlap with other candidate predictors. Status epilepticus was evaluated only when reliably identifiable.

Because only 20 unfavorable outcomes were observed, the primary multivariable logistic regression model was deliberately restricted to two covariates to reduce overfitting and maintain an events-per-variable ratio of approximately 10. Baseline mRS at refractory-state adjudication was included *a priori* as the principal measure of neurological severity. ICU admission was selected as the second covariate because of its clinical relevance as an integrated marker of neurocritical-care requirement and its association with outcome in univariable analysis. Mechanical ventilation was not entered concurrently because of substantial clinical and statistical overlap with ICU admission, with ICU admission retained as the broader critical-care marker. Although age was also associated with outcome in univariable analysis (OR 1.03 per year, P = 0.037), it was not added because this would exceed the prespecified two-variable limit. The final model was therefore interpreted as a parsimonious, clinically guided model rather than a comprehensive variable-selection model. Collinearity between baseline mRS and ICU admission was additionally assessed using variance inflation factors (VIFs), with VIF < 5 considered indicative of no problematic multicollinearity.

For sparse or completely separated variables, descriptive methods and Fisher’s exact test were used where appropriate. All 57 patients had available outcome status, mRS at refractory-state adjudication, and ICU admission and were therefore included in the primary multivariable analysis. Univariable analyses were conducted using available cases for each predictor, and the corresponding analytic denominator was reported. Multiple imputation was not required for variables included in the primary inferential analyses. Variables with substantial missingness, including mechanical ventilation duration and total hospital stay, were excluded from inferential modeling.

Secondary outcomes, including neurological improvement, ICU admission, mechanical ventilation, ICU length of stay, relapse, mortality, and residual deficits, were summarized descriptively as n (%) or median (IQR). ICU length of stay was compared descriptively between outcome groups. Relapse and mortality were reported only as crude documented proportions using the analytic cohort as the descriptive denominator. Because complete individual follow-up durations, censoring times, and reliable event dates were unavailable, these proportions were not interpreted as cumulative 6-month risks, and Kaplan–Meier or Cox regression analyses were not performed. Exploratory analyses compared outcomes according to antibody positivity, anti-NMDAR status, ICU admission, mechanical ventilation, second-line therapy, and maintenance therapy. Formal interaction testing was not performed when event counts were insufficient.

The primary inferential objective was to identify factors associated with unfavorable 6-month functional outcome. Secondary, treatment-related, and subgroup analyses were considered exploratory. Interpretation emphasized the magnitude and direction of associations and their 95% CIs rather than P values alone. No causal conclusions were drawn from treatment-related associations because of the retrospective design, limited sample size, residual confounding, and confounding by indication. Multivariable findings were interpreted cautiously given the limited number of outcome events and the wide confidence intervals.

### 2.6 Ethics

The study protocol, including retrospective medical-record review and structured post-discharge telephone contact when required for outcome ascertainment, was approved by the institutional ethics committee. The ethics committee granted a waiver of written informed consent for both retrospective data collection and minimal-risk telephone outcome ascertainment. Telephone contact did not involve any intervention, treatment recommendation, or additional diagnostic testing and was limited to information necessary for functional-outcome assessment. All source data were de-identified before entry into the study database; direct identifiers (names, ID numbers, contact details, addresses) were removed or replaced with coded identifiers. A locally controlled re-identification file was retained but not included in the final analytic dataset. Data extraction, transfer, storage, and analysis were performed in restricted-access environments. For post-discharge telephone follow-up, investigators verified identity according to institutional procedures and collected only the minimum information needed for outcome adjudication.

## 3 Results

### 3.1 Study screening and baseline characteristics of the study population

A total of 246 potentially eligible records with suspected or documented AE were screened. Forty-eight were excluded during diagnostic review: 31 because the final diagnosis was revised to a non-autoimmune condition and 17 because available evidence was insufficient to satisfy definite/probable AE criteria. Among the remaining 198 diagnostically eligible AE patients, 102 did not fulfill the refractory AE definition after first-line immunotherapy. Of the 96 potentially refractory cases, 39 were further excluded because of major competing systemic conditions (n = 12), insufficient key clinical or treatment data (n = 14), unavailable 6-month functional outcome despite follow-up tracing (n = 8), or duplicate/recurrent episodes excluded after index-episode selection (n = 5). The final analytic cohort therefore comprised 57 patients with refractory AE (**Figure 1**). The demographic characteristics, clinical manifestations, antibody profiles, neuroimaging and EEG findings, cerebrospinal fluid characteristics, critical care requirements, immunotherapy regimens, and short-term outcomes of the overall cohort are summarized in **Table 1**. Study screening and analysis flowchart in **Figure 1**.

**Figure 1:**
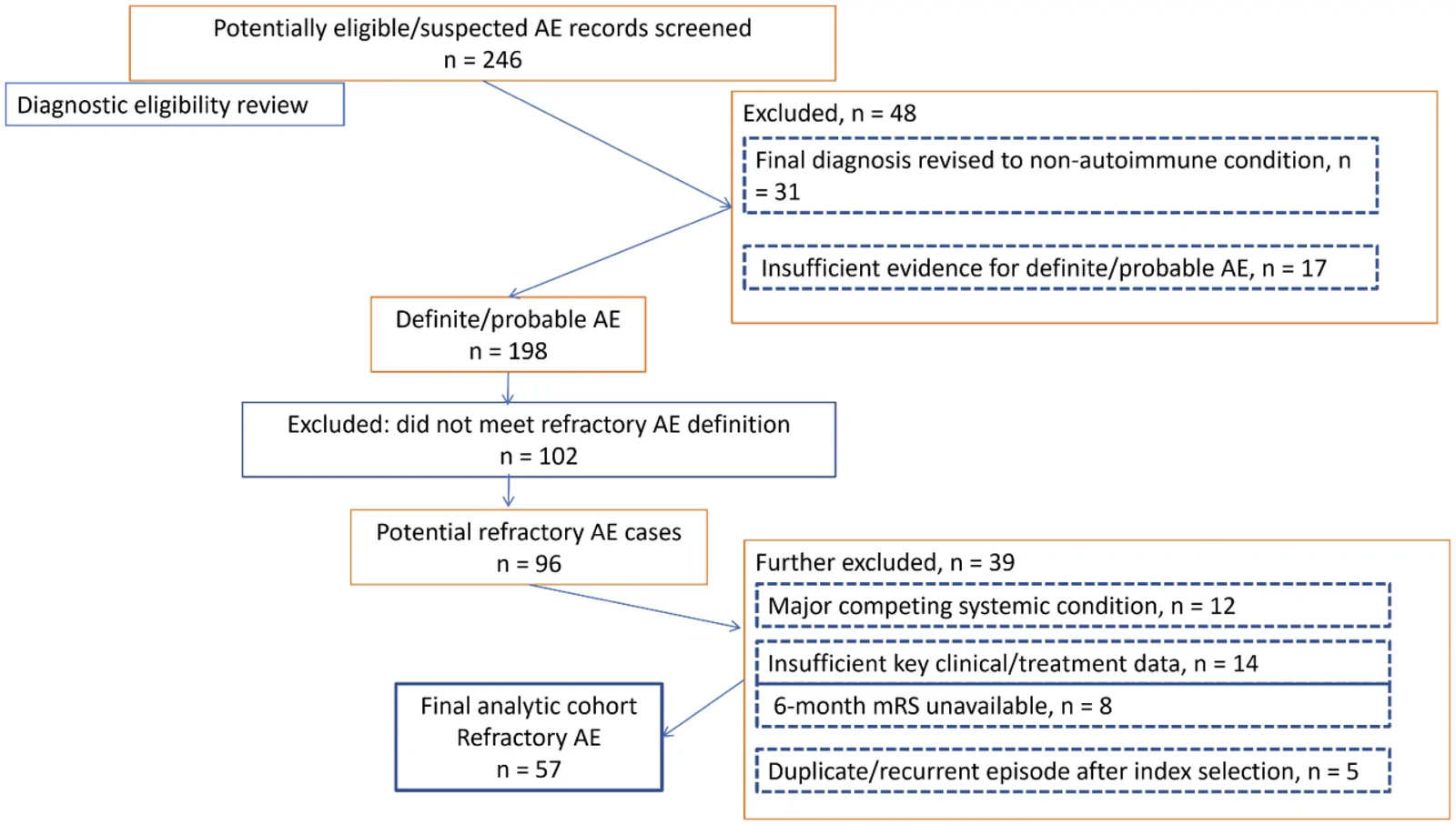
Study screening, eligibility assessment, and analytic cohort flowchart. The flowchart shows sequential diagnostic review, refractory-state eligibility assessment, predefined exclusions, index-episode selection, and derivation of the final 57-patient analytic cohort. AE, autoimmune encephalitis; mRS, modified Rankin Scale; ICU, intensive care unit.

**Table 1:** Demographic, clinical, laboratory, imaging, and treatment characteristics of patients with refractory autoimmune encephalitis (n = 57).

Demographic characteristics	Overall cohort (n = 57)
Age, years, median (IQR)	32 (14–56)
Male sex, n (%)	29 (50.9)
Female sex, n (%)	28 (49.1)
Clinical manifestations	
Prodromal symptoms, n (%)	36 (63.2)
Fever	32 (56.1)
Headache	16 (28.1)
Dizziness	7 (12.3)
Nausea and/or vomiting	9 (15.8)
Cognitive impairment/psychiatric or behavioral abnormalities	39 (68.4)
Seizures	29 (50.9)
Decreased level of consciousness	32 (56.1)
Speech disturbance	14 (24.6)
Involuntary movements	14 (24.6)
Autonomic dysfunction	22 (38.6)
mRS score at refractory-state adjudication, median (IQR)	2 (1–3)
Severe disability at refractory-state adjudication (mRS ≥4)	14 (24.6)
Antibody profiles	
Neuronal/glial antibody positive	24 (42.1)
Anti-NMDAR	12 (21.1)
Anti-LGI1	6 (10.5)
Anti-LGI1/CASPR2 double positive	2 (3.5)
Anti-CASPR2	1 (1.8)
Anti-MOG	2 (3.5)
Anti-GFAP	1 (1.8)
Antibody negative	33 (57.9)
Neuroimaging and EEG findings	
Abnormal brain MRI	50 (87.7)
Normal brain MRI	4 (7.0)
Brain MRI unavailable	3 (5.3)
Abnormal EEG	25 (43.9)
Normal EEG	12 (21.1)
EEG not performed/unavailable	20 (35.1)
Cerebrospinal fluid characteristics	
Pleocytosis/increased CSF cell count	35 (61.4)
Elevated CSF protein	33 (57.9)
Abnormal CSF glucose	5 (8.8)
Positive oligoclonal bands	17 (29.8)
Increased intrathecal IgG synthesis	29 (50.9)
Blood–brain barrier dysfunction	21 (36.8)
Critical care requirements and tumor status	
ICU admission	34 (59.6)
ICU length of stay, days, median (IQR)*	21 (12–27)
Invasive airway support/mechanical ventilation	21 (36.8)
Immunotherapy regimens	
High-dose corticosteroids	52 (91.2)
Intravenous immunoglobulin	44 (77.2)
Plasma exchange	6 (10.5)
Corticosteroids plus IVIG	35 (61.4)
Corticosteroids plus IVIG plus plasma exchange	4 (7.0)
Any second-line immunotherapy	14 (24.6)
Rituximab	9 (15.8)
Cyclophosphamide	3 (5.3)
Ofatumumab	2 (3.5)
Maintenance immunotherapy	10 (17.5)
Mycophenolate mofetil	9 (15.8)
Azathioprine	1 (1.8)
6-month functional outcome	
Favorable outcome (mRS 0–2), n (%)	37 (64.9)
Unfavorable outcome (mRS 3–6), n (%)	20 (35.1)
Death by 6-month outcome assessment, n (%)	1 (1.8)

Death was included within the unfavorable outcome category (mRS = 6) and is shown separately for descriptive purposes only. The exact symptom-onset day was available for 49 patients; in 8 patients (14.0%), only the month of onset was documented and the prespecified monthly midpoint was used for calculation of onset-related time intervals.

After harmonization of the refractory AE definition, all 57 included patients fulfilled the same operational criterion of persistent or progressive neurological dysfunction despite adequate first-line immunotherapy. Second-line therapy was subsequently administered to 14 patients (24.6%), whereas 43 patients (75.4%) were managed without second-line escalation. Maintenance immunosuppression was used in 10 patients (17.5%) as a subsequent longer-term disease-control or relapse-prevention strategy and did not contribute to refractory-state classification.

### 3.2 Short-term functional outcomes and prognostic factors

Among the 57 patients, 37 (64.9%) achieved a favorable short-term functional outcome, while 20 (35.1%) had an unfavorable outcome (mRS 3–6). In univariable logistic regression, older age, higher baseline mRS score, ICU admission, and mechanical ventilation were associated with increased odds of an unfavorable outcome.

In the parsimonious multivariable model including baseline mRS and ICU admission, ICU admission remained associated with an unfavorable 6-month functional outcome after adjustment for baseline mRS (aOR, 5.12; 95% CI, 1.21–21.65; P = 0.026). The wide confidence interval indicates substantial uncertainty in the magnitude of this association. Mechanical ventilation was not entered concurrently because of its substantial overlap with ICU admission. Nevertheless, the adjusted ICU estimate remained imprecise, as reflected by the wide 95% CI, and should therefore be interpreted cautiously. Higher baseline mRS at refractory-state adjudication was also associated with unfavorable outcome (aOR, 1.84 per 1-point increase; 95% CI, 1.05–3.22; P = 0.033). Collinearity diagnostics showed VIFs of 1.42 for both baseline mRS and ICU admission, well below conventional thresholds for problematic multicollinearity. Nevertheless, because baseline mRS and ICU admission may both capture overlapping aspects of underlying disease severity, their coefficients should not be interpreted as representing completely independent prognostic dimensions (**Table 2**).

**Table 2:** Logistic regression analysis of factors associated with unfavorable short-term outcome.

Variable	N analyzed	Univariable OR (95% CI)	P value	Multivariable OR	95% CI	P value
Demographic & Baseline Severity						
Age, per 1-year increase	57	1.03 (1.00–1.05)	0.037	—	—	—
Male	57	1.29 (0.43–3.84)	0.647	—	—	—
mRS at refractory-state adjudication, per 1-point increase	57	2.15 (1.32–3.51)	0.002	1.84	1.05–3.22	0.033
Critical Care Support						
ICU admission	57	11.81 (2.39–58.46)	0.002	5.12	1.21–21.65	0.026
Mechanical ventilation	57	10.00 (2.83–35.31)	<0.001	(Excl. due to collinearity)	—	—
Antibody Profiles & Biomarkers						
Anti-NMDAR	57	0.55 (0.13–2.31)	0.414	—	—	—
Anti-LGI1	57	0.57 (0.10–3.15)	0.523	—	—	—
Anti-CASPR2	57	4.00 (0.34–47.11)	0.271	—	—	—
Anti-GFAP	57	NE	0.351	—	—	—
Anti-MOG	57	NE	0.536	—	—	—
CSF & BBB Indicators						
CSF WBC increase	57	1.27 (0.41–3.92)	0.682	—	—	—
CSF protein increase	57	0.44 (0.15–1.34)	0.151	—	—	—
OCB positive	57	0.29 (0.07–1.17)	0.082	—	—	—
Intrathecal IgG increase	57	1.76 (0.59–5.32)	0.313	—	—	—
BBB dysfunction	57	1.23 (0.40–3.77)	0.716	—	—	—
Immunotherapy Pathways						
Mycophenolate mofetil	57	0.48 (0.09–2.55)	0.386	—	—	—
Azathioprine	57	NE	1	—	—	—

N analyzed represents the number of patients contributing to each univariable analysis. All variables were available for the full analytic cohort (n = 57). The primary multivariable model therefore included all 57 patients. Mechanical ventilation was excluded from the multivariable model because of substantial clinical and statistical overlap with ICU admission. Collinearity between mRS at refractory-state adjudication and ICU admission was low (VIF = 1.42 for both variables). NE, not estimable because of sparse events or complete/quasi-complete separation; OR, odds ratio; CI, confidence interval; mRS, modified Rankin Scale; ICU, intensive care unit; CSF, cerebrospinal fluid; OCB, oligoclonal bands; BBB, blood–brain barrier.

#### 3.2.1 Exploratory analysis of advanced immunotherapy pathways

Treatment pathways involving second-line and maintenance immunotherapy were described exploratorily to characterize real-world management patterns and observed 6-month outcomes. Among the 14 patients who received second-line immunotherapy after refractory-state adjudication, 9 (64.3%) achieved a favorable 6-month functional outcome. Among the 43 patients who fulfilled the same refractory criteria but did not subsequently receive second-line escalation, 28 (65.1%) achieved a favorable outcome (Fisher’s exact P > 0.05). Importantly, the 6-month outcome window in both groups was calculated from the common refractory-state adjudication date rather than from the date of second-line treatment initiation.

For the maintenance phase, 10 patients received mycophenolate mofetil or azathioprine. Favorable 6-month functional outcome was observed in 7/10 patients (70.0%) receiving maintenance immunosuppression and in 30/47 patients (63.8%) who did not receive maintenance therapy. These comparisons were descriptive and hypothesis-generating only. Because treatment allocation was non-random and strongly influenced by disease severity, treatment response, and clinical indication, no conclusions regarding comparative efficacy, equivalence, rescue effects, or treatment-related stabilization can be drawn from these observed proportions.

### 3.3 Functional recovery and critical care outcomes

Among the 57 patients, the median 6-month mRS score was 2.0 (IQR 1.0–3.0). A favorable outcome (mRS 0–2) was achieved by 37 patients (64.9%), while 20 (35.1%) had an unfavorable outcome (mRS 3–6). The distribution of 6-month mRS scores is shown in **Figure 2A**; most patients scored 1 or 2, with fewer having moderate to severe disability (mRS 3–6). Serial assessments were not consistently available, so the temporal course of neurological improvement was not analyzed.

**Figure 2:**
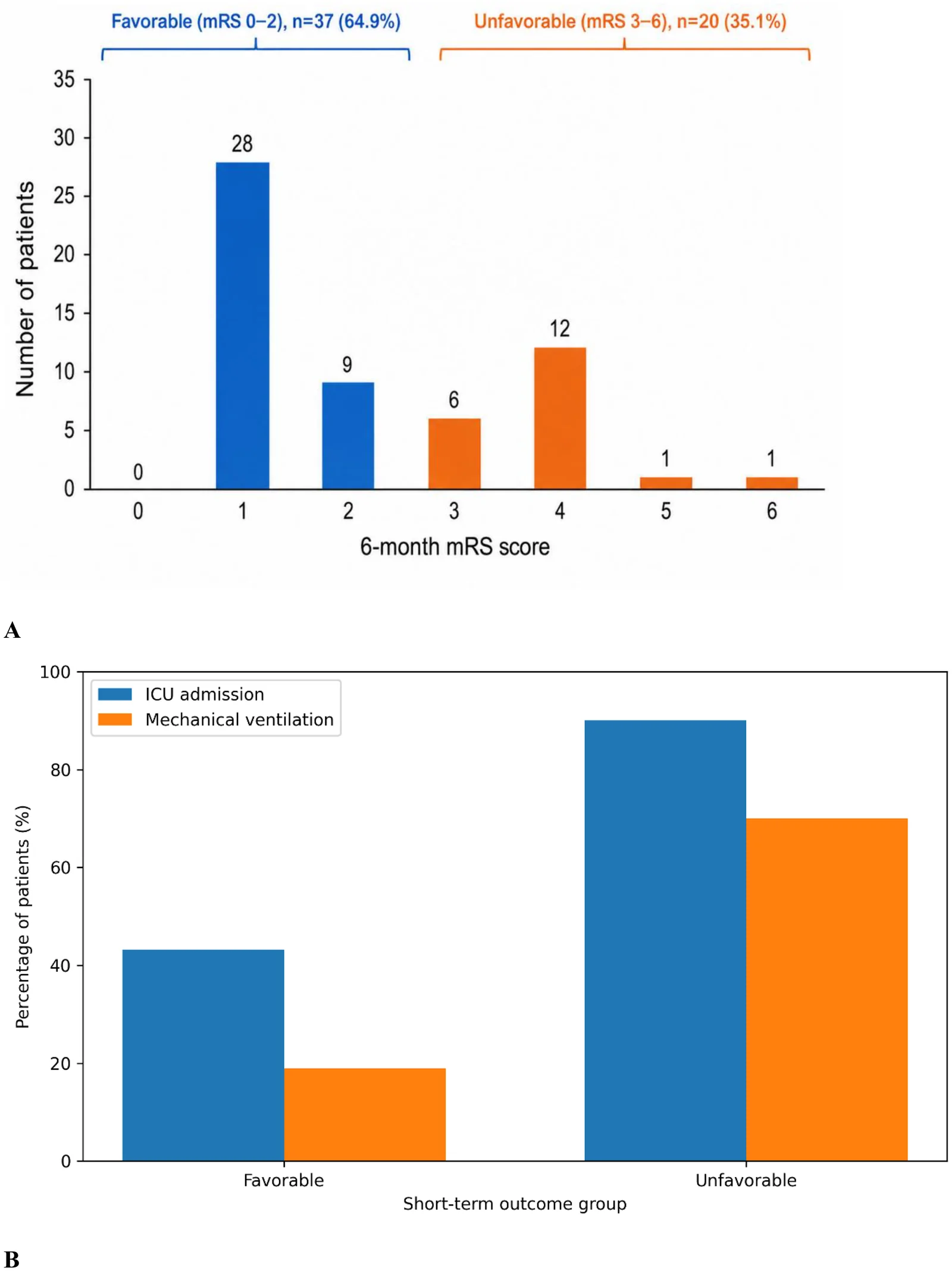
Functional recovery and critical care outcomes in patients with refractory autoimmune encephalitis (n = 57). **(A)** Distribution of short-term functional outcome assessed by the modified Rankin Scale (mRS). Favorable outcome was defined as mRS 0–2, and unfavorable outcome as mRS 3–6. The dashed vertical line indicates the cutoff between favorable and unfavorable outcomes. **(B)** Critical care support according to short-term outcome group. The image compares the proportions of patients requiring ICU admission and mechanical ventilation between the favorable and unfavorable short-term outcome groups.

Critical care requirements differed markedly between outcome groups. ICU admission occurred in 16/37 (43.2%) of the favorable group versus 18/20 (90.0%) of the unfavorable group. Mechanical ventilation was required in 7/37 (18.9%) of the favorable group and 14/20 (70.0%) of the unfavorable group. Among ICU-admitted patients, the median ICU stay was longer in the unfavorable group (21.5 days, IQR 16.3–39.3) than in the favorable group (13.5 days, IQR 11.5–24.0). These findings indicate that poor 6-month functional recovery was associated with greater disease severity and more intensive care support (**Figure 2B**). Data on mechanical ventilation duration and total hospital stay were insufficiently complete and therefore not analyzed.

### 3.4 Relapse and survival

During available post-adjudication follow-up, one verified clinical relapse was documented among the 57 patients (1/57, 1.8%). The event occurred approximately 2 months after discharge in a non-ICU patient with anti-NMDAR encephalitis who had received high-dose corticosteroids without second-line escalation. Because complete individual follow-up durations were not consistently available, the denominator of 57 represents the analytic cohort rather than 57 patients with confirmed relapse-free observation through the entire 6-month period. Accordingly, the observed 1.8% should be interpreted as a crude documented relapse proportion and not as an estimated 6-month relapse risk. One patient (1.8%) died during hospitalization (in the ICU stratum, 1/34, 2.9%); this was an older adult with severe autonomic instability, sustained status epilepticus, and a baseline mRS of 5. No additional relapse or death was identified in the available records, although complete event-free observation through 6 months could not be verified for every patient. Because individual follow-up durations and exact event dates were partially missing, we could not calculate relapse-free or overall survival or perform Kaplan-Meier/Cox analyses. Therefore, these events are reported as crude observed proportions (**Table 3**).

**Table 3:** Summary of relapse, mortality, and in-hospital complications stratified by ICU admission status (n = 57).

Outcome Categories	Overall Cohort (n=57)	ICU Stratum (n=34)	Non-ICU Stratum (n=23)	P value
Survival & Relapse, n (%)				
Documented clinical relapse during available follow-up	1 (1.8)	0 (0.0)	1 (4.3)	0.404^a^
In-hospital Mortality	1 (1.8)	1 (2.9)	0 (0.0)	1.000^a^
Infectious Complications, n (%)				
Respiratory/Pulmonary Infection	31 (54.4)	26 (76.5)	5 (21.7)	< 0.001^b^
Organ System Dysfunction, n (%)				
Hepatic Injury/Dysfunction	15 (26.3)	9 (26.5)*	6 (26.1)*	0.974^b^
Acute Kidney Injury	1 (1.8)	1 (2.9)	0 (0.0)	1.000^a^
Structural Hydronephrosis	1 (1.8)	0 (0.0)	1 (4.3)	0.404^a^
Hematologic Abnormalities, n (%)				
Anemia	7 (12.3)	5 (14.7)*	2 (8.7)*	0.692^a^
Coagulation Disorder	1 (1.8)	1 (2.9)	0 (0.0)	1.000^a^

The relapse percentage uses the full analytic cohort (n = 57) as the descriptive denominator. Because complete individual follow-up durations were not available for all patients, this proportion should not be interpreted as a cumulative 6-month relapse risk. Percentages are calculated within each respective column denominator (57, 34, or 23).

^a^Calculated using Fisher’s exact test due to sparse cell counts or expected frequencies < 5.

^b^Calculated using Pearson’s Chi-square test.

### 3.5 In-hospital complications and safety events

Respiratory or pulmonary infections were the most frequently documented in-hospital complications, occurring in 31 patients (54.4%). CTCAE v5.0 grading was applied retrospectively when sufficient clinical documentation was available; however, incomplete severity-specific documentation prevented uniform formal grading of all infectious and hematologic events. These were heavily concentrated in the ICU stratum (26/34, 76.5%) versus non-ICU (5/23, 21.7%; P<0.001, chi-square). Because pulmonary infections were concentrated among ICU-treated patients and 21 patients required invasive airway support, their occurrence likely reflected a combination of critical illness, airway instrumentation, prolonged hospitalization, and immunosuppressive exposure. The retrospective data did not permit attribution of these infections specifically to immunotherapy. Other complications occurred less frequently: anemia in 7 (12.3%), coagulation disorder in 1 (1.8%); lack of systematic white blood cell nadir logs prevented definitive grading of leukopenia/neutropenia as pure immunotherapy-related toxicity. Hepatic dysfunction (transaminase elevation above institutional thresholds) was noted in 15 patients (26.3%), equally distributed between ICU and non-ICU groups. Renal complications were rare: acute kidney injury in one critically ill patient (1.8%) requiring temporary hemodynamic support, and pre-existing hydronephrosis in another (1.8%). Due to the retrospective design and missing baseline organ function kinetics, hepatic and renal events could not be conclusively attributed to specific therapies (first-line, second-line, or maintenance). Data on mild infusion reactions or premature treatment interruptions were incomplete; therefore, these results represent a pragmatic description of cumulative clinical complications rather than a formal toxicity grading (**Table 3**).

## 4 Discussion

Refractory AE is a highly challenging clinical entity. It is characterized by persistent disease activity, recurrent neuropsychiatric deterioration, or prolonged dependence on neurocritical care despite standard immunotherapy (10, 11). Consensus recommendations have established stepwise treatment pathways. These range from first-line therapies (high-dose corticosteroids, intravenous immunoglobulin, or plasma exchange) to second-line immunosuppression (4, 5). However, real-world evidence on prognostic factors for early recovery in strictly defined refractory populations remains limited (11–13).

In this single-center retrospective cohort, all 57 patients fulfilled a harmonized operational definition of refractory AE based on persistent or progressive neurological dysfunction despite adequate first-line immunotherapy. Subsequent treatment pathways were heterogeneous: 14 patients underwent second-line escalation, whereas 43 did not, allowing treatment escalation to be evaluated separately from the definition of refractoriness itself. We also fitted a parsimonious multivariable model to examine associations of baseline functional severity and critical-care requirement with 6-month functional outcome while limiting model complexity given the small number of unfavorable events. Given only 20 unfavorable events, the model was deliberately restricted to two covariates to reduce overfitting. Baseline mRS was included as the prespecified severity adjustment, and ICU admission was retained as the broader critical-care marker. This approach reduces model complexity but does not eliminate residual confounding or the conceptual overlap between neurological severity and subsequent ICU requirement.

After adjustment for mRS at refractory-state adjudication, ICU admission remained associated with unfavorable 6-month functional outcome (aOR, 5.12; 95% CI, 1.21–21.65). However, the wide confidence interval indicates limited precision, and this finding should not be interpreted as establishing ICU admission as a fully independent dimension of prognosis. Patients with greater neurological severity are inherently more likely to require intensive care; therefore, baseline mRS and ICU admission may partly capture the same underlying severity construct. Although formal collinearity diagnostics did not indicate problematic statistical multicollinearity (VIF = 1.42 for both variables), conceptual overlap in disease severity remains possible. The adjusted association suggests that ICU requirement provides additional prognostic information beyond the measured mRS score, but residual confounding by disease severity cannot be excluded (18, 19).

One possible explanation is the high burden of secondary complications during prolonged neurocritical care. In our cohort, 54.4% of patients had documented respiratory or pulmonary infections. The concentration of pulmonary infections among critically ill patients raises the hypothesis that infection and other ICU-related systemic complications may contribute to impaired neurological recovery. However, infection was not formally evaluated as an independent prognostic factor in the multivariable model, and the present data cannot establish a causal contribution of infection to unfavorable functional outcome (11, 13). This interpretation aligns with findings from Qin et al. and Shan et al. They reported that disorders of consciousness, severe neurological dysfunction, and intensive care requirements were associated with prolonged hospitalization and poor outcomes in severe AE (13, 14). Thus, ICU admission in refractory AE should be viewed not merely as an isolated intervention but as a composite clinical marker. It likely reflects severe neurological involvement together with greater exposure to secondary systemic complications; however, the relative contribution of these factors to functional outcome cannot be separated in this retrospective cohort.

Another pivotal insight comes from our exploratory analysis of real-world advanced immunotherapy pathways. In our cohort, 24.6% of patients received second-line escalation (rituximab, cyclophosphamide, or ofatumumab), and 17.5% received maintenance therapy (mycophenolate mofetil or azathioprine). Evaluating these interventions retrospectively is inherently limited by confounding by indication. Clinicians typically reserve escalation or maintenance for patients with more severe disease, aggressive antibody phenotypes, or poor first-line response (20–22).

Patients who received second-line escalation had a favorable 6-month outcome proportion of 64.3% (9/14), compared with 65.1% (28/43) among those who did not receive second-line therapy. These observed proportions should not be interpreted as evidence of therapeutic equivalence, rescue efficacy, or benefit from treatment escalation. Treatment allocation was non-random, only 14 patients received second-line therapy, and escalation was strongly influenced by clinical severity and response to first-line treatment. Moreover, the counterfactual outcomes of these patients in the absence of escalation cannot be determined. These comparisons should therefore be regarded solely as descriptive and hypothesis-generating (20, 21).

The present cohort was analyzed primarily at the syndrome level because the study objective was to characterize refractory AE encountered in routine clinical practice rather than to estimate subtype-specific treatment effects. However, this approach necessarily pooled biologically and clinically heterogeneous disorders. Thirty-three of 57 patients (57.9%) were antibody-negative, whereas the 24 antibody-positive patients included anti-NMDAR, anti-LGI1, anti-CASPR2, anti-MOG, anti-GFAP, and overlapping antibody phenotypes. These entities may differ substantially in pathophysiology, disease course, relapse risk, treatment responsiveness, and prognosis. Because individual antibody subgroups were small, reliable subtype-specific multivariable analyses were not feasible. Accordingly, the pooled prognostic and treatment-pathway findings should not be assumed to apply uniformly across all AE subtypes.

Several limitations should be acknowledged. First, the retrospective, single-center design precludes causal inference and leaves the possibility of residual confounding. Second, incomplete data prevented consistent assessment of mechanical ventilation duration, total hospital stay, and serial cognitive or psychiatric outcomes. In addition, the exact day of symptom onset was unavailable in 8 patients (14.0%), for whom the midpoint of the documented month was used as a substitute; therefore, time-dependent variables such as onset-to-treatment intervals are subject to measurement uncertainty in these cases. The primary endpoint was limited to the 6-month mRS, which may not fully capture persistent cognitive, sleep, behavioral, or social-function deficits. Some 6-month mRS assessments were also reconstructed from structured telephone interviews rather than direct neurological examinations, introducing potential measurement variability despite use of a standardized follow-up protocol. Third, incomplete follow-up timing and partially unavailable event dates precluded reliable time-to-event analyses. Because complete individual relapse follow-up durations were unavailable, the reported relapse frequency represents only a crude documented proportion and should not be interpreted as a cumulative 6-month relapse risk. Finally, substantial etiologic heterogeneity was present: 33 of 57 patients (57.9%) were antibody-negative, while the antibody-positive subgroup included several distinct AE entities with potentially different natural histories, treatment responses, and prognoses. The small numbers within individual antibody subtypes precluded reliable subtype-specific modeling, and pooled estimates may therefore obscure clinically important between-subtype differences.

The prognostic modeling also has important statistical limitations. A relatively large number of candidate variables were explored despite only 20 unfavorable outcomes, creating potential for multiple-testing effects and unstable univariable estimates. Although the final model was restricted to two covariates to reduce overfitting, ICU admission was selected partly on the basis of its observed univariable association, and baseline mRS and ICU admission may reflect overlapping dimensions of disease severity. The wide confidence interval around the adjusted ICU estimate (aOR 5.12, 95% CI 1.21–21.65) further indicates limited precision. In addition, requiring reliable refractory-state adjudication and ascertainable 6-month outcomes may have introduced selection bias by preferentially retaining patients with more complete longitudinal records. Therefore, the final 57-patient cohort may not fully represent all AE patients treated at the institution, and the multivariable findings should be regarded as exploratory associations requiring validation in larger prospective multicenter cohorts.

## 5 Conclusion

In conclusion, this single-center real-world cohort describes 6-month functional outcomes and prognostic associations in patients with refractory autoimmune encephalitis. A favorable 6-month functional outcome (mRS 0–2) was observed in 64.9% of the cohort. In the parsimonious multivariable model, higher mRS at refractory-state adjudication and ICU admission were associated with unfavorable 6-month functional outcome, although the ICU estimate was imprecise and may partly reflect residual confounding by disease severity. Observed favorable outcome proportions were similar between patients who did and did not subsequently receive second-line therapy, but these treatment-pathway comparisons were descriptive and cannot establish therapeutic equivalence, rescue efficacy, or treatment benefit because of the small sample size and confounding by indication. Maintenance immunosuppression was considered separately as a longer-term disease-control or relapse-prevention strategy, and its observed outcomes were likewise exploratory. Given the etiologic heterogeneity of the cohort and the predominance of antibody-negative disease, these findings should not be assumed to apply uniformly across all AE subtypes. Larger prospective multicenter studies are required to validate these prognostic associations and determine the comparative effectiveness of different treatment pathways.

## Data Availability

The raw data supporting the conclusions of this article will be made available by the authors, without undue reservation.
